# A network system for the prevention and treatment of mushroom poisoning in Chuxiong Autonomous Prefecture, Yunnan Province, China: implementation and assessment

**DOI:** 10.1186/s12889-023-16042-7

**Published:** 2023-10-11

**Authors:** Qunmei Yao, Zhijun Wu, Jiaju Zhong, Chengmin Yu, Haijiao Li, Qiuling Hu, Jianrong He, Jianping Du, Chengye Sun

**Affiliations:** 1 Department of Emergency Medicine, The People’s Hospital of Chuxiong Yi Autonomous Prefecture, Chuxiong, 675000 Yunnan China; 2 National Institute for Occupational Health and Poison Control, Chinese Center for Disease Control and Prevention, Beijing, 100050 China; 3 Chuxiong Yi Minority Autonomous Prefecture Center for Disease Control and Prevention, Chuxiong, 675000 Yunnan China; 4 Chuxiong Health Commission, Chuxiong, 675000 Yunnan China; 5 Dayao People’s Hospital, Dayao, 675400 Yunnan China

**Keywords:** Mushroom poisoning, Fatality, Network, Implementation, Evaluation, Mushroom identification

## Abstract

**Background:**

Mushroom poisoning is a major public health issue in China. The integration of medical resources from different institutes of different levels is crucial in reducing the harm of mushroom poisoning. However, few studies have provided comprehensive implementation procedures and postimplementation effectiveness evaluations. To reduce the harm caused by mushroom poisoning, a network system for the prevention and treatment of mushroom poisoning (NSPTMP) was established in Chuxiong, Yunnan Province, a high-risk area for mushroom poisoning.

**Methods:**

The NSPTMP consists of three types of institutions, namely, centers for disease prevention, hospitals, and health administration departments, with each kind of institution comprising prefecture, county/city, town, and village levels. After three years of implementation, the network was evaluated by comparing the indices before and after network implementation using data from the “Foodborne Disease Outbreak Surveillance System” and 17 hospitals in Chuxiong. The indices included the fatalities caused by mushroom poisoning, the composition ratios of different types of mushrooms for both outpatients and inpatients and the hospitalization rates.

**Results:**

Compared to the average fatality rate of mushroom poisoning from 2015 to 2017, the average fatality rate from 2018 to 2020 significantly decreased from 0.57 to 0.06% (*P* < 0.001). Regarding the poisonous genus containing lethal mushrooms, the outpatient and inpatient composition ratios significantly decreased for *Amanita* (9.36–2.91% and 57.23–17.68%, respectively) and *Russula* (15.27–8.41%) (*P* < 0.05). Regarding poisonous mushrooms that caused mild symptoms, the outpatient and inpatient composition ratios significantly increased for *Scleroderma* (5.13–13.90% and 2.89–18.90%, respectively) and Boletaceae (19.08–31.71%) (*P* < 0.05), and the hospitalization rates significantly increased for *Scleroderma* (6.33–18.02%) and Boletaceae (5.65–12.71%) (*P* < 0.05).

**Conclusions:**

These findings suggest that the NSPTMP effectively reduced the harm caused by mushroom poisoning. In addition to the integration of medical resources, the development of poisonous mushroom identification, hierarchical treatment systems in hospitals, public education, and professional training also played important roles in improving the system’s effectiveness. The establishment and evaluation of the NSPTMP in Chuxiong Prefecture can provide valuable insights and serve as a model for other regions facing similar challenges in managing mushroom poisoning.

**Supplementary Information:**

The online version contains supplementary material available at 10.1186/s12889-023-16042-7.

## Introduction

Mushroom poisoning occurs worldwide [1–3]. In Europe [4–7], the United States [8–10] and China [11–16], the consumption of wild mushrooms is a common practice and often leads to a high number of cases of toxic mushroom exposure. From 1999 to 2016, US poison centers reported 133,700 cases of mushroom poisoning, with 47,220 healthcare facility treatments and 52 deaths [10]. In Germany, from 2010 to 2018, 4441 patients were treated for mushroom poisoning in hospitals, and 32 deaths were recorded from diagnostic data [6]. However, a higher number of mushroom poisoning deaths and fatalities have been observed in China than in Europe and the US. Specifically, from 2004 to 2014, 3710 cases of mushroom poisoning were reported in “The Public Health Emergency Management Information System” in China, with 786 deaths [12]. Recent data from the “Foodborne Disease Outbreak Surveillance System” in China have shown that the proportion of foodborne disease-associated deaths caused by poisonous mushrooms is the highest [17, 18], accounting for 37.78% (51/135), 57.6% (72/125), and 67.86% (76/112) of deaths in 2018, 2019 and 2021, respectively [19–21]. Thus, mushroom poisoning is a major public health problem in China.

Concern about mushroom poisoning in China has been a long-standing issue. Prior to 2000, China had neither a surveillance system that reflected national poisonous mushroom exposure nor a standardized mushroom poisoning prevention and treatment system. In 2000, a mushroom poisoning accident in Guangzhou (9 people were poisoned, and 8 died within a week) [22] sparked concern. Subsequent investigation of this incident revealed that some unique Chinese poisonous mushroom species were misidentified as common species found in Europe or the United States for many years. For example, the mushroom involved in the Guangzhou incident was not the common European species, *Amanita verna*, but a new species that had not yet been identified and was later named by fungal taxonomists *Amanita exitialis* [23]. In 2003, the severe acute respiratory syndrome (SARS) outbreak in China promoted the development of China’s public health system, including the establishment of the “Public Health Emergency Management Information System” by the Chinese Center for Disease Prevention (CCDC) in 2004. This system allows major mushroom poisoning cases to be reported as public health emergency events. At the same time, the surveillance system for foodborne diseases was established in 2001 and updated in 2013. Therefore, with the experience gained from field investigations, phone consultations from poisoning hotlines, and surveillance data analysis, the exposure properties of mushroom poisoning in China gradually became better understood. It became apparent that mushroom identification was crucial in the diagnosis and treatment of mushroom poisoning cases, which is also a consensus revealed in the relevant literature [6, 7, 9, 23]. As a result, mycologists became involved in investigating mushroom poisoning incidents. In 2006, the “Toxic Biological Sample Bank” project was established by the CCDC to obtain the ecological characteristics of poisonous mushrooms and promote the taxonomy of mushrooms in China. Then, books and research papers on poisonous mushroom identification, prevention, and control were published [11, 23–27]. These efforts have shifted the prevention and treatment of mushroom poisoning in China from an empirical mode to an evidence-based mode.

Over the years, experience in preventing, controlling, and treating mushroom poisoning in China has led to the recognition of two key characteristics. First, mushroom poisoning management involves multiple aspects, such as mushroom identification, standardization of clinical diagnosis and treatment, management of local health resources, and public education. Effective management of these aspects requires coordination across various institutions, including hospitals, CDCs, health administration departments (HADs), and research institutes. Second, it has been observed that over 80% of patients with mushroom poisoning experience mild symptoms [7, 10], which suggests that a hierarchal treatment system should be established to ensure that patients with mild symptoms are treated in most healthcare institutions and that patients with severe symptoms are referred to hospitals with life-saving equipment as early as possible. Therefore, the integration of medical resources from different institutes of different levels is crucial in reducing the harm of mushroom poisoning [28, 29]. To address these issues, in 2018, a network system for the prevention and treatment of mushroom poisoning (NSPTMP) was established in the most representative region, Chuxiong Autonomous Prefecture, Yunnan Province [28, 30]. The NSPTMP was designed to reduce the harm of mushroom poisoning in Chuxiong Prefecture with two innovative principles, called “combination and connection”. This means (1) combining the job of prevention at the CDC, the treatment of patients in hospitals, and the authorities of the HAD; and (2) connecting the various levels, from the prefecture, through cities and counties, and down to the townships and villages. After three years of implementation, the effectiveness of the network was evaluated. Although many mushroom poisoning-related studies have proposed strategies to reduce mushroom poisoning risks by strengthening cooperation among various parties and enhancing public education, few have addressed the implementation of such strategies or evaluated their postimplementation effects.

The aim of our study was to reduce the harm caused by mushroom poisoning by establishing the NSPTMP in Chuxiong, Yunnan Province, a high-risk area for mushroom poisoning. This study comprises the following two main components. First, a comprehensive overview of the roles of each kind of institution and their interrelationship at each level within the NSPTMP is presented. Second, the effectiveness of the NSPTMP was evaluated using data from the Foodborne Disease Outbreak Surveillance System and diagnostic data from 17 key hospitals in Chuxiong Prefecture. The findings of this study offer valuable insights for the prevention, control, and management of mushroom poisoning.

## Methods

### Data collection

To assess the impact of the NSPTMP, two distinct data resources were used. First, the Foodborne Disease Outbreak Surveillance System in Chuxiong Prefecture provided insights into mushroom poisoning incidents reported by hospitals across 8 counties and 2 cities in Chuxiong Prefecture (including rural health centers) between 2015 and 2020. Second, data were collected from 17 hospitals within Chuxiong Prefecture, focusing on mushroom species information for patients in both outpatient and inpatient settings.

The surveillance data included the number of individuals who were poisoned and deaths resulting from mushroom poisoning. However, the data offered a general and coarse estimation of the situation, with regional distribution characteristics, but lacked detailed mushroom classification or identification. The hospital data provided a more detailed and specific assessment of mushroom poisoning incidents, helping to paint a more nuanced picture of the impact of the NSPTMP.

### Statistical analysis

The data were analyzed using SPSS version 20.0. The count data are presented as the number of cases or as a percentage and were analyzed using the χ2 test. To assess the impact of the network, comparisons of the indices before and after network implementation were performed. The observed difference was deemed statistically significant at a p value of less than 0.05.

## Results

### Establishment of the NSPTMP in Chuxiong Prefecture, Yunnan Province

**Fig. 1:**
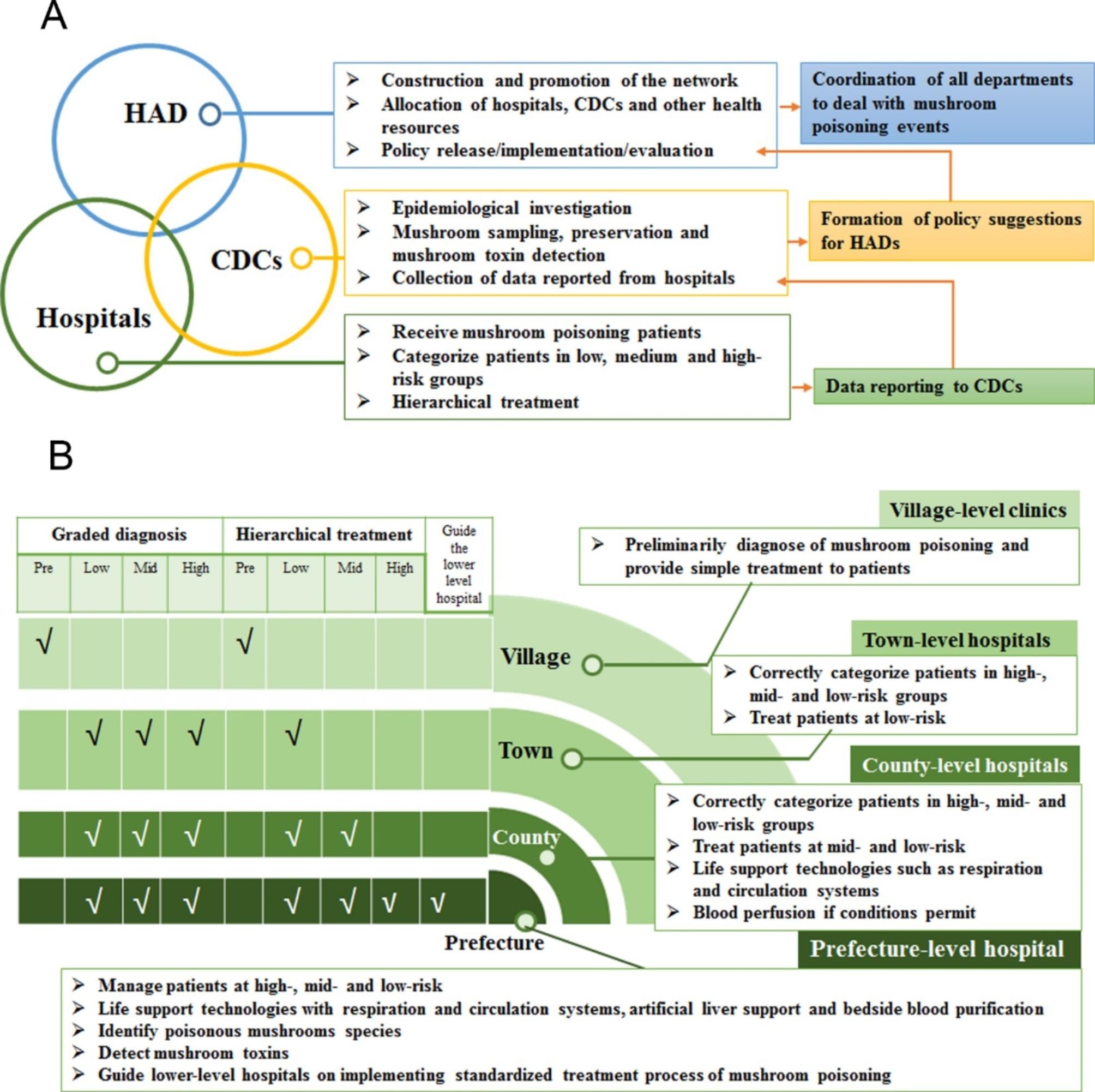
The structure of the NSPTMP. **(A)** Association among three kinds of institutions in the NSPTMP; **(B)** Association of four levels of hospitals in the NSPTMP

The NSPTMP in Chuxiong Prefecture consists of three types of institutions arranged horizontally, namely, CDCs, hospitals, and HADs. This horizontal arrangement serves as the foundation of the “combination” principle (Fig. 1A). Each of these institutions is vertically arranged into four levels, comprising the prefecture, counties/cities, towns, and villages, forming the basis of the “connection” principle, particularly for hospitals (Fig. 1B).

#### HADs at the prefecture, city/county, and town levels

At the prefecture, city/county, and town levels, HADs play a vital role in the NSPTMP. First, HADs can facilitate the development of the NSPTMP and implementation of associated policies based on their organizational structure. Second, HAD authorities are responsible for allocating and coordinating hospitals, CDCs, and other health resources to address mushroom poisoning incidents.

#### CDCs at the prefecture, city/county, and town levels

CDCs operating at the prefecture, city/county, and town levels play a critical role in the NSPTMP. First, CDCs are tasked with conducting field epidemiological investigations, mushroom sampling, preservation, and, in certain cases, mushroom toxin detection (if feasible). Second, in some regions, the establishment of a poisonous mushroom sample library and an online platform for mushroom identification has been initiated to aid in the identification and classification of poisonous mushrooms.

#### Poisonous mushroom sample library

The National Institute of Occupational Health and Poison Control (NIOHP) at the Chinese Center for Disease Control and Prevention (China CDC) initiated a project in 2016 to establish a poisonous mushroom sample library in regions with a high prevalence of mushroom poisoning, such as Guizhou and Yunnan provinces. The goal of this project was to facilitate the standardized collection of mushroom samples by CDCs at all levels in a timely manner. With the successful implementation of this initiative, a searchable poisonous mushroom sample library was established.

#### Online poisonous mushroom identification

During the establishment of the national poisonous mushroom sample library, an effective online working system was developed among doctors, CDC staff, and mycologists, aided by the emergence of new communication tools, particularly the WeChat app. To identify poisonous mushrooms, doctors initially collect clinical symptoms from mushroom poisoning patients. Subsequently, CDC staff collect mushroom samples and upload pictures of these samples to the WeChat group as soon as possible. Third, mycologists conduct a preliminary online identification of the mushroom samples based on the pictures provided, providing feedback to the CDC staff or doctors, which is important information for further diagnosis and treatment. The NIOHP provides final confirmation of the mushroom species by molecular biological identification of mushroom samples sent by local CDCs.

#### Hospitals at the prefecture, city/county, and town levels

Hospitals at various levels, including village, town, county/city, and prefecture levels, exhibit different levels of expertise in recognizing, diagnosing, and treating patients affected by mushroom poisoning.

##### Village-level clinics

: These clinics should have the ability to make preliminary diagnoses of mushroom poisoning and provide simple treatment to patients, with the objective of conducting initial patient grading.

##### Town-level hospitals

: These hospitals should possess the capability to accurately categorize patients into high-, medium-, and low-risk groups and provide treatment for low-risk groups, with the aim of accepting patients with mild symptoms.

##### County-level hospitals

: These hospitals should have the capacity to recognize patients at high, medium, and low risk and provide appropriate treatment to patients in medium- and low-risk groups. Additionally, they should be equipped with life support technologies such as respiration and circulation systems, and when feasible, blood perfusion, to enable treatment of patients with mild to moderate symptoms that are not life threatening.

##### Emergency department of the prefecture-level hospital

: This department should have the capability to manage patients in all risk categories and provide guidance to lower-level hospitals on implementing standardized treatment protocols for mushroom poisoning. Additionally, the department should possess the expertise to identify poisonous mushroom species and detect mushroom toxins. The emergency intensive care unit (EICU) of this hospital should be equipped with advanced life support technologies, including respiration and circulation systems, artificial liver support, and bedside blood purification, to enable effective treatment of patients with complex and serious symptoms, as well as to offer guidance to lower-level hospitals in managing such cases.

### Implementation, practice, and promotion of the NSPTMP

Below, we present a description of the procedures and practical methods of the NSPTMP to enhance residents’ prevention awareness, develop doctors’ diagnostic and treatment standardization skills, and reinforce communication and consensus among relevant professionals.

#### Increasing residents’ awareness of prevention

Given that wild mushrooms are a traditional food in Chuxiong Prefecture, it is challenging for residents to abandon their habit of consuming them. To reduce the likelihood of severe mushroom poisoning, residents are advised against ingesting highly toxic mushrooms, such as *Amanita* and *Russula subnigricans*. If poisoning symptoms arise after consuming wild mushrooms, patients are recommended to seek medical attention immediately. Upon hospital admission, patients are urged to provide a detailed history of mushroom ingestion, along with photographs and mushroom samples whenever feasible to facilitate diagnosis and treatment. To increase public awareness, an advertising video on the clinical prevention and treatment of mushroom poisoning, a sitcom on the classification of diagnosis and treatment of mushroom poisoning, and a popular science exhibition on common poisonous mushrooms in Yunnan Province were produced. During the high-incidence season of mushroom poisoning, these materials are displayed in public gathering areas.

#### Outpatient doctors in hospitals: diagnosis

Physicians diagnose patients with suspected mushroom poisoning based on the *criteria for mushroom poisoning patients* (as follows):

- Patients who provide mushroom pictures or samples, which are identified by experts as poisonous mushrooms;
- Patients whose blood or urine test results show the presence of mushroom toxins detected by high-performance liquid chromatography or enzyme-linked immunosorbent assay;
- Patients with a clear history of poisonous mushroom consumption and corresponding typical poisoning symptoms;
- If the patients or their families are unable to provide pictures or samples of the mushrooms, they can identify the poisonous mushrooms using standard pictures of poisonous mushrooms.

#### Hospital admission/hospitalization/referral: risk classification

In China, mushroom poisoning symptoms include 7 clinical subtypes: acute liver failure, rhabdomyolysis, hemolysis, acute renal failure, gastroenteritis, psycho-neurological disorder, and photosensitive dermatitis, as well as other undetermined clinical types. To achieve the objective of a graded diagnosis, hierarchical treatment, and timely referral for patients, mushroom poisoning is classified into low, medium and high risk levels based on the clinical manifestations and mushroom species. Accordingly, patients can be treated appropriately or transferred to hospitals with better treatment equipment.

- Low-risk group: The patients’ vital signs are stable, and the incubation period is generally less than 6 h. Highly toxic mushroom ingestion has been ruled out. In such cases, possible toxic mushrooms may include Boletaceae (with digestive tract symptoms), *Chlorophyllum*, *Lactarius*, *Russula* (excluding *Russula subnigricans*), *Scleroderma*, *Cordierites frondosus*, and other species.
- Medium-risk group: The patients’ symptoms include transient impaired consciousness, digestive tract symptoms leading to an internal environment disorder, organ function damage, and abnormal liver and kidney function indicators in laboratory examinations. Possible toxic mushrooms may include Boletaceae (which mainly cause neuropsychiatric symptoms), *Amanita* (which may cause neuropsychiatric symptoms or renal damage), and Cortinariaceae.
- High-risk group: The patients’ symptoms are marked by unstable vital signs, impaired consciousness, organ dysfunction or failure, and severe internal environment disorder. Of mushroom poisoning fatalities, 70–90% are caused by acute liver failure induced by mushrooms containing amatoxins, which are primarily found in three genera: *Amanita*, *Galerina*, and *Lepiota. Russula subnigricans* and other mushrooms may also cause fatal poisoning.

#### Highly toxic mushrooms: consensus on diagnosis and treatment

The mushrooms that cause the highest fatality rates among all poisonous mushrooms are those containing amatoxins. In China, the *Consensus on the clinical diagnosis and treatment of poisoning of mushrooms containing amanitin* [31] was coedited by relevant professional committees, the NIOHP in the China CDC, and the People’s Hospital of Chuxiong Prefecture.

#### Hospitals, CDCs, and HAD authorities: communication and training

Communication and training among hospitals, CDCs, and HADs are conducted regularly and include:

- Preparation and practice of the emergency plan for mushroom poisoning.
- Development of a training course on the treatment of mushroom poisoning in hospitals.
- Annually, the People’s Hospital of Chuxiong Prefecture provides further education for 5–10 medical and nursing personnel and offers training on rescue techniques for patients in medium- and high-risk groups for mushroom poisoning. Moreover, the hospital trains county-, town-, and village-level hospitals on the identification and treatment of poisonous mushrooms for mushroom poisoning patients through consultations, counterpart assistance, and medical union systems.
- From 2017 to 2021, the People’s Hospital of Chuxiong Prefecture successfully hosted the Mushroom Poisoning Clinical Prevention and Control Conference annually. The conference attracted scholars from various fields related to mushroom poisoning and provided online and offline training to over 20,000 individuals.

### Evaluation of the NSPTMP by data from the Foodborne Disease Outbreak Surveillance System

Table 1 presents the number of exposures and deaths caused by mushroom poisoning in Chuxiong Prefecture, which includes eight counties and two cities. Overall, between 2015 and 2020, there were 484 reported cases of mushroom poisoning in Chuxiong Prefecture, involving 6,306 individuals, of whom 20 died. After implementing the NSPTMP, although there was no significant change in the total number of mushroom poisoning incidents (3,174 vs. 3,132), the number of fatalities caused by mushroom poisoning substantially decreased (from 18 to 2). Consequently, the fatality rate decreased significantly from 0.57% (before implementation) to 0.06% (after implementation) (*P* < 0.001). Table S1 and Figure S1 in the supplementary materials show the specific number of mushroom poisoning cases in Chuxiong Prefecture with regional information and annual trends.

**Table 1:** Number of mushroom poisonings, deaths and fatalities caused by mushroom poisoning in Chuxiong Prefecture

Time	Number	Deaths	Fatalities (%)
Before implementation (2015–2017)	3174	18	0.57
After implementation (2018–2020)	3132	2	0.06
Total (2015–2020)	6306	20	0.32
χ2			12.618
* P*			< 0.001

### Evaluation of the NSPTMP by data from 17 key hospitals in Chuxiong Prefecture

Between 2015 and 2020, a total of 4,841 patients with mushroom poisoning were admitted to the 17 hospitals we investigated in Chuxiong Prefecture. Among them, 57.34% (2,776/4,841) had information recorded regarding the identification of the mushroom species involved. This section only presents data on patients with documented species identification of poisonous mushrooms ingested.

Comparing the total number of outpatient and inpatient cases of mushroom poisoning before (2015–2017) and after the implementation of the NSPTMP (2018–2020), there was a relatively stable number of outpatients (1,539 vs. 1,237) and inpatients (172 vs. 164). However, the composition ratios of recorded mushroom species exhibited a significant change in both outpatient and inpatient cases.

Table 2 shows the changes in the composition ratios of recorded mushroom species among outpatient and inpatient cases of mushroom poisoning before and after the implementation of the NSPTMP. The data indicate that after the implementation of the NSPTMP, there was a significant decrease in the outpatient composition ratio regarding highly toxic *Amanita* mushroom poisoning from 9.36 to 2.91% (*P* < 0.001), as well as a decrease in the outpatient composition ratio for *Russula* mushrooms (probably with highly toxic species) from 15.27 to 8.41% (*P* < 0.001). Similarly, there was a decrease in the inpatient composition ratio for *Amanita* mushroom poisoning from 57.23 to 17.68% (*P* < 0.001).

However, there were also changes in the composition ratios of nonlethal mushroom poisonings. The proportion of *Scleroderma* mushroom poisoning in outpatients increased from 5.13 to 13.90% (*P* < 0.001), and the composition ratio of Boletaceae mushroom poisoning in inpatients increased from 19.08 to 31.71% (*P* = 0.008). The composition ratio of *Scleroderma* mushroom poisoning inpatients also increased from 2.89 to 18.90% (*P* < 0.001).

**Table 2:** Mushroom species composition for outpatients and inpatients (number of patients, %)

	Mushrooms with highly toxic genera			Nonlethal mushrooms					Others or unknown			Total
	*Amanita*	*Russula* ^1^		Boletaceae	*Scleroderma*	*Lactarius*	*Cordierites frondosus*		Others^2^	Unknown		
Outpatient												
Before	144(9.36%)	235(15.27%)		584(37.95%)	79(5.13%)	83(5.39%)	19(1.23%)		8(0.52%)	387(25.15%)		1539(100%)
After	36(2.91%)	104(8.41%)		409(33.06%)	172(13.90%)	64(5.17%)	24(1.94%)		15(1.21%)	413(33.39%)		1237(100%)
Total	180(6.48%)	339(12.21%)		993(35.77%)	251(9.04%)	147(5.30%)	43(1.55%)		23(0.83%)	800(28.82%)		2776(100%)
χ^2^	46.999	3.123		7.117	64.115	0.003	2.239		4.006	22.705		
*P*	< 0.001	< 0.001		0.080	< 0.001	0.957	0.135		0.045	< 0.001		
Inpatient												
Before	99(57.23%)	9(5.20%)		33(19.08%)	5(2.89%)	2(1.16%)	10(5.78%)		4(2.44%)	10(5.78%)		172(100%)
After	29(17.68%)	4(2.44%)		52(31.71%)	31(18.90%)	4(2.44%)	6(3.66%)		4(2.44%)	34(20.73%)		164(100%)
Total	128(38.10%)	13(3.87%)		859(25.30%)	36(10.71%)	6(1.79%)	16(4.76%)		8(2.38%)	44(13.10%)		336(100%)
χ^2^	56.604	1.761		6.965	22.453	0.222	0.860		0.000	16.417		
*P*	< 0.001	0.184		0.008	< 0.001	0.638	0.354		1.000	< 0.001		

Note: ^1^The genus *Russula* includes lethal *Russula subnigricans*; ^2^Others include *Trogia venenata*, *Lepiota*, and *Chlorophyllum molybdites*

After the implementation of the NSPTMP, there was no significant change in the hospitalization rate for highly toxic mushroom poisoning (*Amanita* and *Russula*), while the hospitalization rate for nonlethal mushroom poisoning (Boletaceae and *Scleroderma*) significantly increased. Table 3 shows that the hospitalization rate for Boletaceae mushroom poisoning increased significantly from 5.65% (33/584, before implementation) to 12.71% (52/409, after implementation, *P* < 0.001), and the hospitalization rate *for Scleroderma* mushroom poisoning increased significantly from 6.33% (5/79, before implementation) to 18.02% (31/172, after implementation, *P* = 0.014).

**Table 3:** The hospitalization rates for different poisonous mushroom species

	Hospitalization rate
Mushrooms with highly toxic genera *Amanita*	
Before	68.75%(99/144)
After	80.56%(29/36)
χ^2^	1.954
*P*	0.162
*Russula*	
Before	3.83%(9/235)
After	3.85%(4/104)
χ^2^	0
*P*	1
Nonlethal mushrooms	
Boletaceae	
Before	5.65%(33/584)
After	12.71%(52/409)
χ^2^ *P*	15.332 < 0.001
*Scleroderma*	
Before	6.33%(5/79)
After	18.02%(31/172)
χ^2^	6.026
*P*	0.014
*Cordierites frondosus*	
Before	52.63%(10/19)
After	25%(6/24)
χ^2^	3.465
*P*	0.063
*Lactarius*	
Before	2.41%(2/82)
After	6.25%(4/64)
χ^2^	1.361
*P*	0.243

## Discussion

Various types of data resources reflect exposure to poisonous mushrooms, including health surveillance systems [6, 7, 10], hospital visit records [7, 32], field surveys [11], telephone or online consultations [9, 13–15, 33], and literature reviews. Data from surveillance systems in China show that mushroom poisoning is one of the leading causes of death by foodborne poisoning. Over the past 20 years, as research and the understanding of mushroom poisoning in China have improved, it gradually became apparent that integrating the efforts of the CDC, hospitals, and HADs at various levels is a necessary step in reducing the harm of mushroom poisoning. In 2018, the NSPTMP was established in Chuxiong Prefecture, Yunnan Province, a representative high-risk area for mushroom poisoning in China, based on the principles of “combination and connection”. In this paper, we provided a comprehensive introduction to the NSPTMP and evaluated its effectiveness before and after its implementation. The evaluation showed that the implementation of the NSPTMP was effective in reducing the harm caused by mushroom poisoning, as evidenced by the following: (1) The total number of mushroom poisoning cases was almost stable before and after its implementation (3174 vs. 3132), but the number of mushroom poisoning deaths decreased from 18 to 2, with a significant decrease in the fatality rate (0.57% vs. 0.06%, *P* < 0.001). (2) After the implementation of the NSPTMP, the composition ratio for lethal mushrooms decreased significantly for outpatients (*Amanita* and *Russula*) and inpatients (*Amanita*), suggesting that residents reduced the consumption of mushrooms that can cause serious illness. (3) After the implementation of the NSPTMP, the composition ratios of mushrooms that caused minor symptoms significantly increased for both outpatients (*Scleroderma*) and inpatients (Boletaceae and *Scleroderma*), indicating that with the decrease in highly toxic mushroom poisoning, mushrooms that caused mild symptoms received more attention.

The effectiveness of the NSPTMP is based on the development and standardization of the following three aspects: identification of poisonous mushrooms, hierarchical treatment in hospitals, and public education and professional training.

Identifying mushroom species is crucial for clinical diagnosis and hierarchical management. In China, research on the identification of poisonous mushrooms began relatively late compared to Europe and the United States. However, over the past two decades, Chinese scholars have conducted systematic research on poisonous mushrooms, and more than 520 species of poisonous mushrooms have been reported. The toxins of highly toxic poisonous mushrooms in China differ substantially from those of mushrooms in other countries. For example, the amatoxin levels in *Amanita exitialis*, unique to China, are 2–5 times higher than those in *Amanita verna*, a species common in Europe [34, 35]. The poisonous mushroom identification rate in China has gradually increased, with 57% (2776/4841) of the mushroom poisoning cases received by 17 hospitals in Chuxiong Prefecture undergoing mushroom species determination from 2015 to 2020, compared to only 0.93% of mushroom poisoning events with mushroom specimen collection and identification from 2010 to 2014, as reported in the surveillance system [12]. However, the literature shows that the correct identification rate of mushroom species in mushroom poisoning events is quite low, ranging from 5 to 27% [2, 7, 36–39]. To improve identification accuracy, the basis for mushroom species determination has evolved from identification by pictures to standardized collection and preservation of mushroom samples and morphological and molecular biological identification. Additionally, research on mushroom taxonomy has promoted the detection of mushroom toxins and toxicity research of whole mushrooms or their toxins, which are of great value for toxin determination in clinical biological samples, poisoning mechanism research, and therapeutic drug development.

Hierarchical treatment in hospitals is an essential component of the NSPTMP. Although mushroom poisoning is a major public health issue, most cases are mild, and only a small proportion are severe and require hospitalization [10, 32]. In Chuxiong Prefecture, between 2015 and 2020, of the 2776 outpatient cases of mushroom poisoning in 17 hospitals, the majority were poisoned by nonlethal mushrooms, such as Boletaceae, *Scleroderma*, and *Lactarius*, with Boletaceae being the most common (993 cases/2776 cases, 35.77%). Therefore, correct identification of mushrooms is crucial for appropriate hospital surveillance and management [7, 36–38]. To address this, the NSPTMP adjusts medical resources based on diagnosis by grade and hierarchical treatment, resulting in more high-risk group patients being admitted by the People’s Hospital of Chuxiong Prefecture (Table S1). In 2021, the Yunnan Provincial Health Commission Office adopted the strategy of diagnosis by grade and hierarchical treatment” for the diagnosis and treatment of mushroom poisoning patients.

The NSPTMP also emphasizes public education and professional training. Regarding public education, some studies have shown that residents’ misrecognition of edible mushroom species is the most common cause of poisoning, which can be prevented through science popularization and public education [10]. As the consumption of delicious wild mushrooms is a way of life in Yunnan, it is impossible to persuade residents not to eat wild mushrooms at all. The NSPTMP focuses on the popular science work regarding highly toxic mushrooms by warning against the consumption of *Amanita* and *Russula*, which might be associated with the decrease in lethal mushroom consumption reflected by hospital outpatient records. Second, for professional training, the NSPTMP organizes regular training for medical staff, including the identification of mushroom species, clinical diagnosis, and treatment of mushroom poisoning. In addition, the program also provides training for village doctors, who are usually the first responders to mushroom poisoning cases in rural areas. Through training and education, medical staff and village doctors can improve their knowledge and skills in the diagnosis and treatment of mushroom poisoning, which can ultimately improve the quality of care for patients and reduce the fatality rate of mushroom poisoning cases. The NSPTMP also collaborates with local communities and stakeholders to raise awareness of the risks associated with wild mushroom consumption, promoting safe and responsible mushroom foraging practices to prevent mushroom poisoning.

However, there were still some limitations in our study. First, there was still a certain proportion of mushrooms listed as “unknown” mushroom species in the analysis of the mushroom species composition for outpatients (800/2776, 28.82%) and inpatients (44/336, 13.10%). It is necessary to analyze the causes of these “unknown” mushroom classifications in the future and identify more new species if possible. Second, we used only three years before and after the implementation of the NSPTMP to evaluate the NSPTMP, and more data need to be accumulated in the future to support the effectiveness of the NSPTMP.

Importantly, although mushroom poisoning may have regional characteristics, it is still a global public health issue. Some countries may lack reliable mushroom poisoning reporting systems and related death registries, and the risk of mushroom poisoning may be considerably underestimated. Studies have shown that in addition to China, other countries, such as Russia, Ukraine, Belarus, Poland, Turkey, Iran, Nepal, and Mexico, have high fatality rates from mushroom poisoning [1]. The establishment and evaluation of the NSPTMP in Chuxiong Prefecture can provide valuable insights and serve as a model for other regions facing similar challenges in managing mushroom poisoning.

## Conclusions

In this paper, a network system for the prevention and treatment of mushroom poisoning was established in a high-risk area for mushroom poisoning, Chuxiong. The evaluation of the NSPTMP suggested that the network can effectively reduce the harm caused by mushroom poisoning. Overall, the successful implementation of the NSPTMP in Chuxiong Prefecture highlights the importance of a comprehensive and coordinated approach to public health issues. By adopting the principles of “combination and connection” and the three core foundations of the NSPTMP (identification of poisonous mushrooms, hierarchical treatment in hospitals, and public education and professional training), other regions can develop effective strategies for the prevention and control of mushroom poisoning. However, it is crucial to adapt these strategies to the local context, accounting for differences in the types of poisonous mushrooms, the availability of medical resources, and the cultural practices related to mushroom consumption.

### Electronic supplementary material

Below is the link to the electronic supplementary material.

Supplementary Material 1

## Data Availability

The data will not be shared publicly; however, the data are available upon reasonable request. If someone wishes to request the data from this study, author Qunmei Yao (18987838279@163.com) can be contacted.

## Abbreviations

NSPTMP: Network system for the prevention and treatment of mushroom poisoning
CDC: Center for Disease Control
HAD: Health administration department
NIOHP: National Institute of Occupational Health and Poison Control
EICU: Emergency intensive care unit
