# Supplementary material for: A network system for the prevention and treatment of mushroom poisoning in Chuxiong Autonomous Prefecture, Yunnan Province, China: implementation and assessment
Source: BMC Public Health. 2023 Oct 11;23:1979. doi: 10.1186/s12889-023-16042-7 (PMC10568813; doi:10.1186/s12889-023-16042-7)
Supplement: Supplementary file 1 — Supplementary Material 1 [file 12889_2023_16042_MOESM1_ESM.docx]

**Supplementary materials**

**A network system for the prevention and treatment of mushroom poisoning in Chuxiong Autonomous Prefecture, Yunnan Province, China: Implementation and assessment**

Qunmei Yao^1,*^, Zhijun Wu^2,*^, Jiaju Zhong^1^, Chengmin Yu^1^, Haijiao Li^2^, Qiuling Hu^3^, Jianrong He^4^, Jianping Du^5^, Chengye Sun^2,†^

^1^Department of Emergency Medicine, The People’s Hospital of Chuxiong Yi Autonomous Prefecture, Chuxiong, Yunnan, China, 675000;

^2^National Institute for Occupational Health and Poison Control, Chinese Center for Disease Control and Prevention, Beijing, China, 100050;

^3^Chuxiong Yi Minority Autonomous Prefecture Center for Disease Control and Prevention, Chuxiong, Yunnan, China, 675000;

^4^Chuxiong Health Commission, Chuxiong, Yunnan, China, 675000;

^5^Dayao People’s Hospital, Dayao, Yunnan, China, 675400;

^*^These authors contribute equally to the work and are co-first authors of the article

^†^Corresponding authors. Chengye Sun, Email: suncy@chinacdc.cn, 86-10-83132660

Table S1 Regional distribution of Mushroom Poisoning population in Chuxiong Prefecture (from 2015 to 2020)

| Region (city/county) | Residential population (%) | Mushroom poisoning | |  | Deaths caused by mushroom poisoning | | |
| --- | --- | --- | --- | --- | --- | --- | --- |
|  |  | Number | Composition ratio (%) |  | Number | Composition ratio (%) | fatality (%) |
| Chuxiong | 26.13 | 1863 | 29.54 |  | 12 | 60.00 | 0.64 |
| Lufeng | 15.17 | 702 | 11.13 |  | 1 | 5.00 | 0.14 |
| Wuding | 9.89 | 697 | 11.05 |  | 0 | 0.00 | 0.00 |
| Dayao | 9.47 | 503 | 7.98 |  | 1 | 5.00 | 0.20 |
| Nanhua | 8.43 | 414 | 6.57 |  | 3 | 15.00 | 0.72 |
| Yuanmou | 8.34 | 428 | 6.79 |  | 1 | 5.00 | 0.23 |
| Yao'an | 6.79 | 441 | 6.99 |  | 0 | 0.00 | 0.00 |
| Mouding | 6.18 | 577 | 9.15 |  | 1 | 5.00 | 0.17 |
| Shuangbai | 5.54 | 175 | 2.78 |  | 0 | 0.00 | 0.00 |
| Yongren | 4.05 | 506 | 8.02 |  | 1 | 5.00 | 0.20 |
| Total | 100.00 | 6306 | 100.00 |  | 20 | 100.00 | 0.32 |

As Table S1 shows, the composition ratio of mushroom poisoning population in each region is close to that of residential population in each region, with the difference within 4%. Chuxiong (city) has the largest number of mushroom poisoning population (1893 cases, accounting for 29.54%), which may be related to its highest composition ratio of its residential population (26.13%). The number of deaths from mushroom poisoning in Chuxiong (city) is also the largest (12 deaths), which may be related to that more high-risk group patients admitted by the People's Hospital of Chuxiong Prefecture.


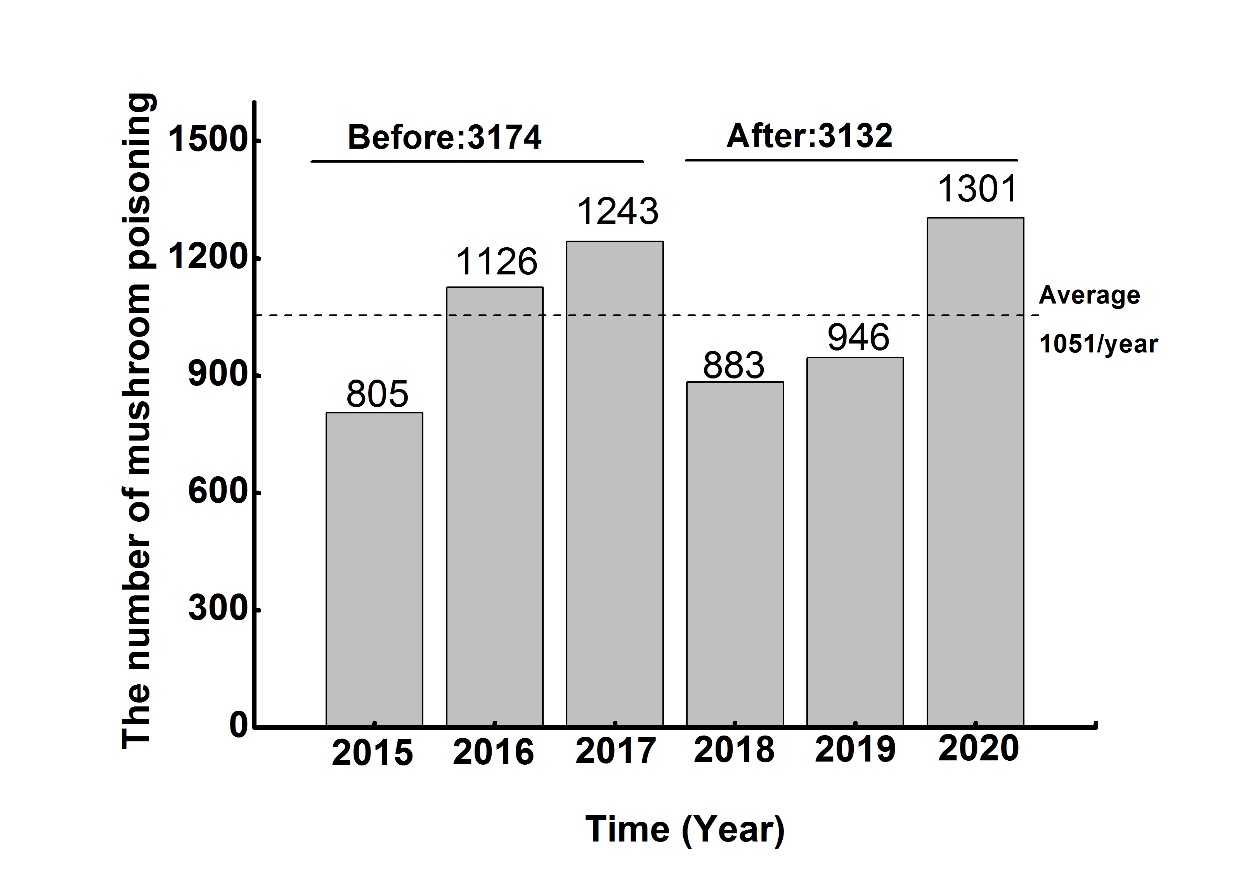


Figure S1 Annual change trend of the total number of mushroom poisoning in Chuxiong Prefecture (2015-2020)
